# The complete mitochondrial genome of *Isonychia kiangsinensis* (Ephemeroptera: Isonychiidae)

**DOI:** 10.1080/23802359.2018.1467233

**Published:** 2018-04-27

**Authors:** Qi-Meng Ye, Shu-Sheng Zhang, Yin-Yin Cai, Kenneth B. Storey, Dan-Na Yu, Jia-Yong Zhang

**Affiliations:** a College of Chemistry and Life Science, Zhejiang Normal University, Jinhua, China Zhejiang;; b Zhejiang Wuyanling National Nature Reserve, Taishan, China;; c Key Lab of Wildlife Biotechnology, Conservation and Utilization of Zhejiang Province, Zhejiang Normal University, Jinhua, China;; d Department of Biology, Carleton University, Ottawa, Canada

**Keywords:** Ephemeroptera, mitochondrial genome, *Isonychia kiangsinensis*, phylogeny

## Abstract

The complete mitochondrial genome of *Isonychia kiangsinensis* is a circular molecule of 15,456 bp in length, containing 2 rRNA genes, 13 protein-coding genes, 22 tRNA genes, and a control region. The AT content of the overall base composition is 62.9%. The length of the control region for *I. kiangsinensis* is 745 bp with 68.6% AT content. In BI and ML phylogenetic trees, *Isonychia kiangsinensis* was a sister clade to *I. ignota* and Isonychiidae was shown to be the basal clade of Ephemeroptera excluding Siphluriscidae. The monophyly of the families Isonychiidae, Heptageniidae, Viemamellidae, and Baetidae and the genus *Isonychia* were well supported.

The family Isonychiidae is composed of one genus (*Isonychia*) and two subgenera (*Isonychia* and *Prinoides*) (Tiunova et al. 2004; Tungpairojwong and Boonsoong 2011). The phylogenetic relationship of Isonychiidae is controversial both in morphological and molecular aspects (Demoulin 1961; McCafferty and Edmunds 1979; Hebert et al. 2003; Ogden and Whiting 2005; Sun et al. 2006; O’Donnell and Jockusch 2008; Ogden et al. 2009; Webb et al. 2012; Saito et al. 2016). More molecular evidence needs to be discovered to clarify the status of this system. Thus, we sequenced the mitochondrial genome of *Isonychia kiangsinensis* and discussed its phylogenetic relationship within Ephemeroptera.

Samples of *I. kiangsinensis* were collected in Jingning (27°58′22′′ N, 119°38′10′′ E), Zhejiang province, China and identified by Dr. Zhang. The total genomic DNA was extracted from the hindleg of *I. kiangsinensis* using an Ezup Column Animal Genomic DNA Purification Kit (Sangon Biotech Company, Shanghai, China). All mayflies samples and DNA samples were stored in the lab of Dr. Zhang, College of Chemistry and Life Science, Zhejiang Normal University. The universal primers and specific primers for polymerase chain reaction (PCR) amplification were designed as in Zhang et al. (2008).

The mitochondrial genome of *I. kiangsinensis* showed the typical insect arrangement and is a circular molecule of 15,456 bp length. The AT content of the overall base composition is 62.9%, and the length of the control region is 745 bp with 68.6% AT content. Most of the protein-coding genes (PCGs) used ATN (N represents A, T, C, G) as the initiation codon whereas *ND2* and *ND5* were initiated by GTG. The *COX1*, *COX2*, *ND4*, *ND5*, and *Cyt b* genes used T as the termination codon and the other PCGs ended with TAA or TAG.

Bayesian inference (BI) and maximum likelihood (ML) trees were constructed using the 13 PCGs from 22 species (Zhang et al. 2008; Li et al. 2014; Tang et al. 2014; Zhou et al. 2016; Gao et al. 2018) including *Siphluriscus chinensis* (Li et al. 2014) as the outgroup (Figure 1). To select conserved regions of the nucleotides, each alignment was performed by Gblocks 0.91b (Castresana, 2000). BI and ML analyses were performed by MrBayes 3.1.2 (Huelsenbeck & Ronquist, 2001) and RAx ML 8.2.0 (Stamatakis 2014), respectively.

**Figure 1.**
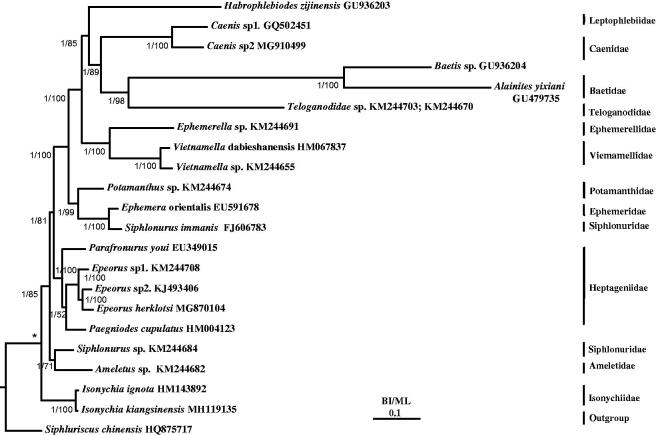
Phylogenetic tree of the relationships among 22 species of Ephemeroptera, including *Isonychia kiangsinensis* based on the nucleotide dataset of the 13 mitochondrial protein-coding genes. Numbers above branches specify posterior probabilities as determined from BI (left) and bootstrap percentages from ML (right). The GenBank accession numbers of all species are also shown.

*Isonychia kiangsinensis* was shown to be a sister clade to *I. ignota* (HM143892). *Siphluriscus chinensis* (Siphluriscidae) is the basal clade to Ephemeroptera and Isonychiidae is the basal clade to Ephemeroptera excluding Siphluriscidae. The monophyly of the families Isonychiidae, Heptageniidae, Viemamellidae, and Baetidae and the genus *Isonychia* were well supported in both BI and ML analyses (Figure 1). The monophyly of Siphlonuridae failed to be supported in BI and ML analyses as also reported by Gao et al. (2018). Long branch attraction was found in Baetidae which may affect the phylogenetic relationship between Teloganodidae and Baetidae. In this study, Teloganodidae is a sister clade to Baetidae (*Baetis* sp. + *Alainites yixiani*) as also shown in Gao et al. (2018) but differs from the results of Ogden and Whiting (2005).

## Nucleotide sequence accession number

The complete mitochondrial genome of *Isonychia kiangsinensis* has been assigned the GenBank accession number MH119135.
